# Renewal of radiological equipment

**DOI:** 10.1007/s13244-014-0345-1

**Published:** 2014-09-18

**Authors:** European Society of Radiology (ESR)

**Affiliations:** Neutorgasse 9/2, 1010 Vienna, Austria

**Keywords:** Radiological equipment, Obsolescence, Risks, Renewal

## Abstract

In this century, medical imaging is at the heart of medical practice. Besides providing fast and accurate diagnosis, advances in radiology equipment offer new and previously non-existing options for treatment guidance with quite low morbidity, resulting in the improvement of health outcomes and quality of life for the patients. Although rapid technological development created new medical imaging modalities and methods, the same progress speed resulted in accelerated technical and functional obsolescence of the same medical imaging equipment, consequently creating a need for renewal. Older equipment has a high risk of failures and breakdowns, which might cause delays in diagnosis and treatment of the patient, and safety problems both for the patient and the medical staff. The European Society of Radiology is promoting the use of up-to-date equipment, especially in the context of the EuroSafe Imaging Campaign, as the use of up-to-date equipment will improve quality and safety in medical imaging. Every healthcare institution or authority should have a plan for medical imaging equipment upgrade or renewal. This plan should look forward a minimum of 5 years, with annual updates.

**Teaching points**

• Radiological equipment has a definite life cycle span, resulting in unavoidable breakdown and decrease or loss of image quality which renders equipment useless after a certain time period.

• Equipment older than 10 years is no longer state-of-the art equipment and replacement is essential. Operating costs of older equipment will be high when compared with new equipment, and sometimes maintenance will be impossible if no spare parts are available.

• Older equipment has a high risk of failure and breakdown, causing delays in diagnosis and treatment of the patient and safety problems both for the patient and the medical staff.

• Every healthcare institution or authority should have a plan for medical imaging equipment upgrade or replacement. This plan should look forward a minimum of 5 years, with annual updating.

## Introduction

Medical imaging has a crucial role in modern healthcare systems. It is almost impossible to appropriately diagnose and treat most health conditions without the use of state-of-art imaging equipment.

## Evolution in medical imaging

Imaging equipment is high-tech material requiring advanced electronic and mechanical engineering, together with a functional design. It is a very active research sector. Decade by decade progress in technology offers considerable improvement in terms of quality and security, both for diagnostic and therapeutic imaging.

Increase in spatial and temporal resolution, combined with a better lesion characterisation, leads to identification and diagnosis of smaller lesions, with considerable impact on patient care, e.g. in cancer. Interestingly, innovation in imaging technologies results in a better imaging quality while also improving security. Cardiac CT imaging is a typical example of the evolution towards lower radiation exposure levels while improving lesion conspicuity; the newest technology offers 10–30% the radiation exposure levels of systems 5 years ago. Based on this, newer technology, even though it is being used more, will lead to a reduced overall medical radiation exposure of the community.

Besides providing fast and accurate diagnosis, advances in radiology equipment offer new and previously non-existing options for treatment guidance with quite low morbidity, resulting in improvement in health outcomes and quality of life for patients.

## Equipment life cycles

The radiological equipment has a definite life cycle span, resulting in unavoidable breakdown and decrease or loss of image quality, which renders equipment useless after a certain time period [1–3]. The state of the equipment is also affected by its utilisation and maintenance.

The Canadian Association of Radiologists endorses general rules regarding the life cycle of various types of equipment based on their utilisation, which is categorised into three categories (high, mid and low) on the basis of number of exams per year, as shown in Table 1 [2].

**Table 1:** Medical imaging equipment life expectancy guidance (utilisation and age related)

Device type (analogue or digital)	Device life expectancy based on utilisation: HIGH-MID-LOW	Utilisation based on exams/year		
		HIGH	MID	LOW
Radiography, general	10-12-14	>20,000	10,000-20,000	<10,000
Radiography, mobile	10-12-14	>6,000	3,000-6,000	<3,000
R/F fluoroscopy (conventional/remote)	8-10-12	>4,000	2,000-4,000	<2,000
R/F interventional integrated c-arm	8-10-12	>4,000	2,000-4,000	<2,000
R/F urology	8-10-12	>1,500	750-1,500	<750
Mobile C-arm (all types including O-Arms)	8-10-12	>2,000	1,000-2,000	<1,000
Angiography (1/2 plane)/interventional	8-10-12	>4,000	2,000-4,000	<2,000
Cardiac suite (single/biplane)	8-10-12	>3,000	1,500-3,000	<1,500
CT scanner	8-10-12	>15,000	7,500-15,000	<7,500
MRI scanner	8-10-12	>8,000	4,000-8,000	<4,000
Ultrasound	7-8-9	>4,000	2,000-4,000	<2,000
SPECT/gamma	8-10-12	>6,000	3,000-6,000	<3,000
SPECT/CT	8-10-12	>4,000	2,000-4,000	<2,000
PET (likely replace with a different technology such as PET/CT)	8-10-12	>6,000	3,000-6,000	<3,000
PET/CT	8-10-12	>4,000	2,000-4,000	<2,000
Bone densitometry	8-10-12	>10,000	5,000-10,000	<5,000
Mammography	8-9-10	>7,000	3,500-7,000	<3,500
Lithotripter	8-10-12	>3,000	2,000-3,000	<2,000

*HIGH* 24 h/day 5 days/week or 750 8-h shifts/year, *MID* 16 h/day 5 days/week or 500 8-h shifts/year, *LOW* 8 h/day 5 days/week or 250 8-h shifts/year

In Table 1 an examination is a defined technical investigation using a medical imaging modality to study a body structure, system or anatomical area that yields one or more views for diagnostic and/or therapeutic purposes. Exceptions include routinely ordered multiple body structures that by common practice or protocol are counted as one exam. Hence, the examination of one body region without and with contrast medium counts as one exam, and the number of exams increases accordingly when two or more body regions are examined. The figures for cardiac suite and angiographic procedures do not specify the ratio of diagnostic versus interventional procedures [2].

As older equipment has a high risk of failure and breakdown, this may lead to crucial delays in the diagnosis and treatment of the patient. Moreover older equipment might cause safety problems both for the patient and for the medical staff [2–4]. Operating costs of older equipment will be high when compared with new equipment and sometimes maintenance will be impossible if no spare parts are available. Technical or functional obsolescence might deteriorate the functionality of radiology equipment.

The European Society of Radiology (ESR) is promoting the use of up-to-date equipment, especially in the context of the EuroSafe Imaging Campaign, as the use of up-to-date equipment will improve quality and safety in medical imaging [5]. It is known that equipment is up to 5 years old reflects the current state of technology and offers opportunities for economically reasonable upgrade measures. Equipment which is between 6 and 10 years old is still fit to use if properly maintained, but already requires replacement strategies to be developed. Equipment older than 10 years is no longer state-of-the art equipment and replacement is essential. It is recommended that at least 60% of the installed equipment in radiology departments should be up to 5 years old. Up to 30% should be 6–10 years old, whereas not more than 10% of equipment should be older than 10 years [6].

The age profile of radiology equipment hardware is not the only factor presenting the state-of-art status. Technological advances render some equipment obsolete, or require software and hardware upgrades to keep the existing equipment in the state-of-art status or simply in a satisfactory status for a certain period of time. But after a certain age, upgrade and even repair become no longer possible [2, 3]. Therefore equipment becomes progressively under-used and discarded, serving at best as a source for spare parts. Another limit of old equipment is its inability to be included in an up-to-date communicating environment which requires a performing electronic infrastructure. Examples are telemaintenance, teleradiology, patient identity propagation and connection with the electronic patient record.

## Economic considerations

Modern healthcare is very competitive, and patients and healthcare authorities are demanding the best for the patient in most European countries. This can only be achieved by the use of state-of-art imaging technologies [7].

However, imaging equipment is very expensive to install and maintain. The constrained healthcare budgets create dilemma almost in all countries and practice in Europe is very diverse regarding the renewal of radiology equipment, as the consequence of considerable differences among healthcare systems in different states, percentage of GDP allocated for healthcare in specific countries, reimbursement policies in specific countries, regions and institutions, and many other factors, such as access rationalisation and equipment use optimisation.

In some countries (especially those with National Health Service types of centralised healthcare and universal coverage of population), austerity and efficiency policies are severely restricting the available finances for capital equipment. Also, the low or decreasing reimbursement for imaging procedures due to economic difficulties and saving policies results in longer periods of use of specific radiology equipment as it becomes harder or impossible for specific departments to obtain new equipment from the responsible health authorities, or as the cycle of return on investment is extended. This severely affects the whole health sector, both private and public, including academic departments, particularly in those countries—within the EU or outside of it—that are/were severely affected by the economic crisis. The fact that older equipment is used would eventually result in higher costs due to the delay in diagnosis and treatment, and increasing maintenance costs. However, specific data about these economic figures are lacking, to our knowledge, but undoubtedly many departments have a considerable proportion of equipment in use that is in need of immediate replacement [8].

## Drivers for equipment renewal

### Local decisions

Local decisions rely on combination of multiple criteria: age, breakdowns and availability rate, operational costs, repair possibilities, medical benefit of the technology, functionality as regards the clinical requirements, image quality, safety (radiation), risk of claims, regulatory obligations, equipment efficiency (ergonomics, patient throughput), strategic factors such as attractiveness for the employees and patients [9, 10].

Experts (radiologists and biomedical engineers) should develop control on image quality and raise alarm bells when appropriate care of patients is no longer offered. In parallel, users should prospectively gather precise data on equipment malfunction (number of hours of partial or total failure) and try to estimate its consequences (appointment delays, underemployment of human resources) [10].

### General incentive measures

Health policies in some countries have set up different incentives leading to equipment quality improvement and transparency towards both requesters and patients. Examples of such measures are:

- Regulatory obligation of specific mentions in the medical report about the type and age of the equipment used, the radiation dose for technologies using X-rays, all these data being indirect information to assess the quality of the examination
- Regulatory obligations to measure and optimise the radiation dose
- Quality control of the equipment (security, image, radiation)
- Reimbursement models on fees for exam, taking into account the level of performance of the equipment, its age and possible upgrade [11]

However, specific regulations in different European countries vary or sometimes do not exist at all.

## Recommendations

Every healthcare institution or authority should have a plan for medical imaging equipment upgrade or renewal. This plan should look forward a minimum of 5 years with annual updating. Studies have shown that the lifetime of medical equipment will be prolonged by up to 50% if it is not utilised often compared with institutions with high utilisation rates of the equipment. Also, if maintenance is ignored, equipment lifetime will be shortened by up to 50%. Another consideration will be the changes in medical practice which will affect the need for some types of imaging [2, 7, 8, 10, 12].

Within an environment where decisions are mainly driven by financial considerations, business models should include the global cost for running equipment and not purely the acquisition cost. Decisions should intelligently take into consideration not only immediate results but also the cost of poor quality, errors and diagnostic delays [13, 14].

The ESR strongly promotes the use of up-to-date equipment also in the context of the EuroSafe Imaging Campaign, as the use of up-to-date equipment will improve quality and safety in medical imaging [5]. The ESR’s general position is that equipment which is up to 5 years old has state-of-art of technology. Properly maintained equipment which is between 6 and 10 years old is still suitable for use. However, a replacement strategy has to be developed. If equipment is older than 10 years, it is not accepted as state-of-the art equipment and replacement is essential.
